# Nearly complete mitochondrial genome of *Siobla xizangensis* Xiao, Huang & Zhou, 1988 (Hymenoptera: Tenthredinidae) and phylogenetic analysis 

**DOI:** 10.1080/23802359.2019.1692729

**Published:** 2019-11-20

**Authors:** Xi Luo, Meicai Wei, Gengyun Niu

**Affiliations:** College of Life Sciences, Jiangxi Normal University, Nanchang, China

**Keywords:** Mitogenome, gene rearrangement, phylogeny, Tenthredinidae, *Siobla*

## Abstract

In this study we review the phylogenetic grouping of the sawfly genus *Siobla* within the superfamily Tenthredinoidea. Using Next Generation Sequencing (NGS), we describe the complete mitochondrial genome of *Siobla xizangensis* Xiao, Huang & Zhou, 1988. The assembled mitochondrial genome of *S. xizangensis* was found to be 15015 bp, with three tRNA genes rearranged compared to the ancestral organization. The overall nucleotide composition was 42.6% for A, 11.4% for C, 7.7% for G and 38.3% for T. The phylogenetic tree based on heterogeneity models of 36 Symphytan and two Apocritan recovered the monophyly of Tenthredinidae, and *S. xizangensis* was identified as the sister group of *Tenthredo*.

Systematic studies of the genus *Siobla* (Hymenoptera, Tenthredinoidea, Tenthredinidae, Sioblinae) have recently been performed (Niu and Wei 2010). Within the genus, a range of morphological characteristics were reported according to the species groups (Niu et al. 2012; Niu and Wei 2013). However, significant inconsistencies with the position of *Siobla* within the superfamily Tenthredinoidea have been reported. Here, we describe the nearly complete mitochondrial genome of *S. xizangensis* Xiao et al. 1988 to advance our understanding of the phylogenetic status of *Siobla* amongst the Tenthredinoidea.

Samples of *S. xizangensis* were collected in Mêdog 52 K, Tibet Autonomous Region (29.74°N 95.68°E) in 2019. The specimens (CSCS-Hym-MC0150) used to obtain the samples were taken are available at the Asia Sawfly Museum, Nanchang (ASMN) repository. Whole genomic DNA was extracted from the specimen’s thorax muscle using the DNeasyR Blood & Tissue Kits (Qiagen, Valencia, CA). Genomic DNA was sequenced by the high-throughput Illumina Hiseq 4000 platform, yielding a total of 42756724 raw reads (SRR10207561). DNA sequences were assembled using MitoZ (Meng et al. 2019), and Geneious Prime 2019.2.1 (https://www.geneious.com) using *S. sturmii* (unpublished) as reference, the mean coverage is 21135. Annotations were generated in MITOS web server (Bernt et al. 2013) and revised in Geneious Prime when necessary.

Upon initial attempts, the nearly complete mitochondrial genome of *S. xizangensis* was found to be 15015 bp in length and accessible on GenBank (accession number MN562486). Furthermore, the analysis yielded a genome containing 37 genes, including 13 protein-coding genes (PCGs), 22 transfer RNA genes and two ribosomal RNA (rRNA) genes. The ancestral pattern of A + T rich region-*trnI*(+)-*trnQ*(-)-*trnM*(+) clusters were rearranged to *trnQ*(-)-*trnM*(-)- A + T rich region -*trnI*(+). There are five gene overlaps among *atp8-atp6* (7 bp), *atp6-cox3* (1 bp)*, trnE* and *trnF* (2 bp)*, nad4-nad4L* (4 bp) *and nad6-cob* (1 bp). There are 14 gene intervals among *nad2*-*trnW* (14 bp), *trnC*-*trnY* (1 bp), cox*2*-*trnK* (3 bp), *cox3*-*trnG* (6 bp), *nad3*-*trnA* (4 bp), *trnA*-*trnR* (4 bp), *trnR*-*trnN* (16 bp), *trnS1*-*trnE* (3 bp), *trnF*-*nad5* (2 bp), *trnH-nad4* (6 bp), *nad4L*-*trnT* (2 bp), *trnP*-*nad6* (2 bp), *trnS2*-*nad1* (7 bp), *trnQ*-*trnM* (4 bp). The *rrnS* and *rrnL* genes were 809 bp and 1334 bp in length, respectively, and located between *trnL1* and *trnQ*, separated by *trnV*. The base composition was 34.8% (A), 10.3% (C), 9.9% (G) and 44.9% (T), with a high A + T content (79.8%) of PCGs. The length of the PCGs accounted for 74.9% of the mitogenome. Only *cox1* start with GTG, the remainder PCGs start with typical ATN codon. *cox1* and *cob* are terminated with incomplete stop codon T, and the remaining PCGs with the stop codon TAA.

The eleven unsaturated amino acids (*atp8* and *nad4L* were excluded) of 36 Symphytan and two Apocritan were aligned in TranslatorX (Abascal et al. 2010) subjected to Bayesian analysis with PhyloBayes (Lartillot et al. 2009) under the MtArt-CAT model conducted on the CIPRES (Miller et al. 2010) webserver (Figure 1). All related files have been uploaded to figshare (https://figshare.com/account/home#/projects/70835). *S. xizangensis* was identified as the sister group of *Tenthredo.*Furthermore, phylogeny inference under the heterogeneous model confirmed that *Athalia* was a basal lineage amongst the Tenthredinoidea (He et al. 2019), and that Pamphiliidae and Megalodontesidae are recovered as monophyletic Pamphilioidea (Niu et al. 2019).

**Figure 1.**
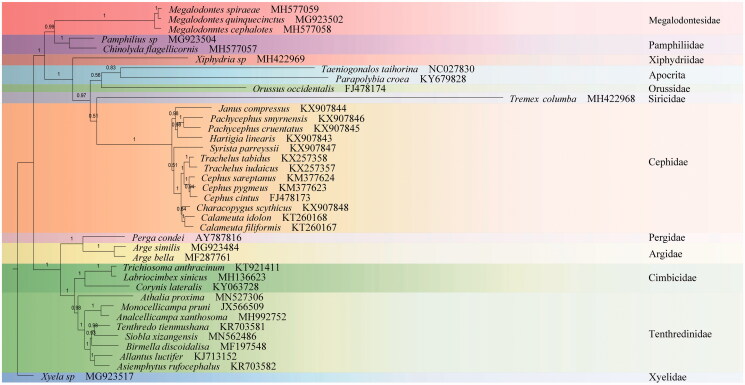
Phylobayes tree based on the combined data of eleven unsaturated amino acids. Numbers above each node are posterior probabilities. The accession number for each species is indicated after the Latin name.
